# Oleogels, Bigels, and Emulgels: Fabrication, Application and Research Trends

**DOI:** 10.3390/gels11100816

**Published:** 2025-10-12

**Authors:** Cristina Ghinea, Ana Leahu

**Affiliations:** Faculty of Food Engineering, Stefan cel Mare University of Suceava, 720229 Suceava, Romania; analeahu@fia.usv.ro

## 1. Introduction

Gels are created by entrapping liquid oil (oleogels) or water (hydrogels) into the well-organized three-dimensional network of a gelling agent [1]. Mixing oleogels and hydrogels in different proportions forms hybrid gels or bigels [1,2], while emulsion and gel mixtures can form emulgels [3].

Oleogels are a healthier alternative to saturated and trans fats [4], with applications in many areas of the food industry, such as bakery [5,6], meat [7,8], confectionary [9,10], dairy [11,12], etc. Oleogels can also be used in the pharmaceutical industry as carriers for nutraceuticals and in cosmetics for the delivery of lipophilic compounds or in dermatological products targeting skin barrier problems [13], while hydrogels have demonstrated significant environmental benefits in wastewater treatment [14]. Bigels also have applications in the food industry, but these have been under-investigated in the literature, compared to pharmaceutical applications, where they are mainly used as a drug delivery vehicle, particularly in transdermal applications [15]. Emulgels have been investigated as fat replacers (e.g., a margarine alternative in sponge cakes) in the production of functional foods [16] or for the treatment of various skin disorders [17].

The primary aim of this Special Issue, “Oleogels, Bigels, and Emulgels: Fabrication, Application, and Research Trends”, is to investigate recent advances and current knowledge in the study of different gel types in the food and cosmetic industries, dentistry (i.e., for treating infected root canals), and water management. This Special Issue comprises nine contributions (seven original research articles and two review articles) that address thematic investigations into the preparation and use of gels as follows: as inks for 3D food printing technology, for replacing solid fats in cookies and sausages, in skin care applications, in antibacterial therapies for root canal disinfection, and for water control under extreme reservoir conditions.

## 2. Overview of the Publications in This Special Issue

In “Design of Aerated Oleogel–Hydrogel Mixtures for 3D Printing of Personalized Cannabis Edibles”, Andriotis et al. (contribution 3) developed and investigated 3D printable edible inks by mixing oleogel and hydrogel phases. They used oleogels from cannabis seed oil and xanthan gum hydrogels mixed with air (via a syringe coupling method) to prepare these inks. The visual appearance and flow rate of different ink formulations, as well as printability, were evaluated. The rheological properties of different ink formulations, the microstructure of the inks, and the thermal behavior during heating were also investigated. The results of this study contribute to 3D food printing technology, posing a method to introduce new ingredients into the diet and a possible solution to food insecurity [18].

The second study, “Effect of Cooling Rate on Properties of Beeswax and Stearic Acid Oleogel Based on Rice Bran Oil and Sesame Oil” by Sivakanthan et al. (contribution 4), explored the effects of cooling rates on the rheological properties, microstructure, and oil-binding capacity of oleogels. Oleogels were obtained via direct oleogelation from sesame oil–rice bran oil and beeswax–stearic acid mixtures. Their results showed that the oil-binding capacity was higher for oleogels that cooled more slowly. Also, rheological properties showed that slow cooling yields more desirable properties in the beeswax–stearic acid oleogel compared to rapid cooling rates. The authors stated that the higher oil-binding capacity could be attributed to the larger fractal dimensions of oleogels cooled at a slower rate. The results obtained from this research present potential for the food industry, especially in manipulating and improving oleogel properties.

The third study, “Textural, Color, and Sensory Analysis of Cookies Prepared with Hemp Oil-Based Oleogels”, by Leahu et al. (contribution 5), examined the rheological and textural profile of cookies formulated with hemp seed oil oleogels. The oleogelation agents used were beeswax, candelilla wax, carnauba wax, sitosterol, pea protein, and xanthan gum. In this work, the potential of oleogels as solid fat substitutes in cookies was investigated. The results showed increased hardness of cookie doughs with oleogels and of baked products. The colors of the cookie samples were different depending on the oleogel used; for example, the color of those with natural wax and hemp seed oil is darker, with a red and yellow hue. Investigating the use of different gelators for oleogels could have practical applications as a replacement for solid fats.

In “Injectable and Conductive Polyurethane Gel with Load-Responsive Antibiosis for Sustained Root Canal Disinfection”, Mu et al. (contribution 6) developed injectable conductive polyurethane gels incorporating piezoelectric n-BaTiO_3_ as a sustained bacteriostasis strategy for root canal therapy. The synthesis and characterization of conductive polyurethane-based prepolymers, physicochemical properties of injectable conductive polyurethane gels for root canal therapy, characterization of electrochemical properties of cured gels, bacteriostasis properties of cured gels, in vitro biofilm eradication and bacterial killing efficiency of cured gels, and others were discussed in this paper. This study provides a significant advance in active response therapeutic systems for prolonged infection control in root canal therapy.

The following article, “Development of Liposome-Based Hydrogel Patches Incorporating Essential Oils of African Plants and Deep Eutectic Solvents” by Jiang et al. (contribution 7), introduced liposome-based hydrogel patches that integrate deep eutectic solvent, argan oil, and passion fruit seed oil. The authors use liposomal encapsulation to stabilize the deep eutectic solvent (composed of betaine and phytic acid) against hydration-induced degradation and synergy with essential oils to enhance transdermal permeability in advanced skin care applications. Spectral and chromatographic analysis of betaine-phytic acid, analysis of the essential oil’s properties (pH, density, apparent viscosity, percentage of free radical scavenging capacity), and characterization of nanoliposome structure and properties were performed. Nanoliposome-loaded hydrogel patches were also investigated and characterized. Their results showed that the hydrogel has exceptional hydration properties, which can improve skin texture and appearance. Also, colloidal stability and transdermal permeability were significantly improved by encapsulating bioactive oils in nanoliposomes.

In “Phytochemical Characterization, Bioactivities, and Nanoparticle-Based Topical Gel Formulation Development from Four Mitragyna speciosa Varieties”, Anantaworasakul et al. (contribution 9) developed topical gels based on nanoparticles from ethanolic extracts of four varieties of kratom for cosmeceutical skincare applications. The kratom nanoparticle formulations were characterized based on particle size and polydispersity index, as well as zeta potential values. Moreover, phytochemical analysis, antioxidant activities (DPPH, ABTS, and FRAP), and collagenase and elastase inhibitory activities were investigated. The results of Anantaworasakul et al.’s study (contribution 9) revealed that kratom nanoparticle-loaded gels had a brownish appearance, with good clarity and the uniform dispersion of nanoparticles within the gel. The gels obtained in this work seem suitable for skin application, as they have an acceptable pH, constant viscosity, and stable appearance.

The article “In Situ-Prepared Nanocomposite for Water Management in High-Temperature Reservoirs”, by Yang et al. (contribution 8), presented the development of a hydrogel system via in situ crosslinking of polyacrylamide with phenolic resin, reinforced with silica sol nanoparticles, for application in water control in high-temperature reservoirs. The authors investigated improvements in gelation performance, thermal stability, water retention, strengthening mechanism, and relevant field performance. They determined that a gelation period of 72 h at 130 °C was optimal for the gelation system. Thus, under these conditions, a plugging efficiency of 92.4% was achieved. Finally, their results outline a practical solution for water control under extreme reservoir conditions that require delayed gelation and sustained mechanical performance.

This Special Issue also includes two comprehensive review articles that synthesize recent advances in the fabrication, application, and research trends of oleogels, bigels, and emulgels. The first review, “Food-Grade Bigel Systems: Formulation, Characterization, and Applications for Novel Food Product Development” by Zampouni et al. (contribution 1), described recent advances in the development of the two structured phases (oleogels and hydrogels) of bigels and their use in various food applications. The authors discussed the types and characteristics of oleogelators, oils, and hydrogels. They also presented the bigel preparation techniques (cold and hot homogenization) and the most used characterization techniques for bigel systems (physical properties, microstructure, textural and rheological properties, thermal properties, swelling capacity, and stability). Bigels can be used as delivery systems for bioactive compounds (lycopene, lutein, β-carotene, quercetin, fatty acids from vegetable oils), as fat replacers in meat and bakery products, or in 3D food printing and other applications, such as edible coatings.

The second review, “Exploring the Feasibility of Direct-Dispersion Oleogels in Healthier Sausage Formulations” by Mahmud et al. (contribution 2), evaluated the potential use of oleogels in healthier sausage formulations. The authors presented a brief history and current size of the sausage market, the health risks of sausage consumption, and market trends in the production of healthier sausages. Incorporating oleogels into sausages may be a solution to the challenges of low-fat and low-saturated fat sausages. This review presents and outlines various direct dispersion oleogels for sausage manufacturing applications, the incorporation of oleogels in the mixing step of sausage processing, oleogel mechanisms, emulsion stability and slice-ability, cooking yields, and the effects of oleogels on nutritional, textural, and sensory properties of sausages. By carefully optimizing formulation, processing, and equipment to maintain consistent quality and functionality, oleogels could be industrially applied in sausage manufacturing to meet public health objectives. But rigorous quality control measures, optimization of formulation, processing, and equipment, cost efficiency, regulatory compliance, and appropriate labeling will be required.

## 3. Conclusions

The research and reviews presented in this Special Issue highlight the methods of preparation, analysis, and use of different types of gels in the food industry and beyond. Innovation starts from gel compositions (kratom nanoparticle-loaded gels; cannabis seed oil, hemp seed oil, sesame oil, and rice bran oil oleogels; xanthan gum hydrogel; hydrogel system with polyacrylamide, phenolic resin, reinforced by silica sol nanoparticles; encapsulation of *Argania spinosa* and *Passiflora edulis* essential oils in hydrogel; polyurethane gels incorporating piezoelectric n-BaTiO_3_) and selection of preparation methods (like cold and hot homogenization techniques for bigel preparation), and continues with the characterization of gels to understand and optimize their properties (gelation and application performances; antioxidant activities; appearance, microstructure, swelling, and mechanical properties of hydrogel patches; antibacterial properties of gels; oil-binding capacity and rheological properties of oleogels, etc.). The microstructure, mechanical, and thermal properties and stability of gels should be determined before their use in food applications to ensure desired functionality and consumer acceptance. The uniform distribution of oleogels in the food matrix, with the preservation of the gel structure under different conditions, as well as the replication of the sensory and textural characteristics of animal fats by oleogels, represents a key challenge in the food industry. Furthermore, long-lasting skin hydration by hydrogels is crucial for skin care applications. Investigating the antibacterial properties of gels under pressure-induced activation for root canal therapy is particularly important in dentistry for more effective and dynamic long-term disinfection compared to traditional static methods. Extended gelation time, high thermal stability, effective operational efficiency, and gel degradability are critical for enhanced oil recovery in high-temperature reservoirs, particularly for water control.

The future of gels is very promising in the food, cosmetic, pharmaceutical, dental, and environmental sectors, due to their unique properties. However, much more detailed research is needed. The full potential of gels can be unlocked through collaborative interdisciplinary research, essential for the transition from laboratory experiments to industry; therefore, engineers, chemists, biologists, and clinicians should work together.
